# Problematic internet use: A growing concern for adolescent health and well-being in a digital era

**DOI:** 10.7189/jogh.14.03034

**Published:** 2024-08-30

**Authors:** Abel Fekadu Dadi, Berihun A Dachew, Gizachew A Tessema

**Affiliations:** 1 Menzies School of Health Research, Charles Darwin University, Northern Territory, Australia; 2 Addis Continental Institute of Public Health, Addis Ababa, Ethiopia; 3 Curtin School of Population Health, Curtin University, Perth, Western Australia, Australia; 4 Shool of Public Health, University of Adelaide, Adelaide, South Australia, Australia

Common mental health problems represent the largest burden of disease among young people. Globally, over one in seven adolescents suffer from mental health and behavioural disorders. Suicide is the fourth leading cause of death in this age group with more than 80% of these deaths occurring in low and middle-income countries (LMICs) [1]. While the causes of mental illness in young people are multifactorial [2] – encompassing genetic predisposition, environmental stressors, social influences, biological changes, psychological factors, and lifestyle choices – the interaction of these factors with their constant connectivity to digital media is increasingly evident to have a negative impact [3,4]. Although digital media offers a sense of connection and various benefits, excessive use can paradoxically heighten feelings of isolation and loneliness, particularly when it replaces face-to-face interaction and real-world engagement [5]. For example, adolescents with a genetic predisposition might be more susceptible to the negative impacts of digital media, such as cyberbullying or exposure to harmful content, which can trigger or worsen their mental health issues [6]. Digital media can amplify societal norms that glorify certain body images or lifestyles, contributing to adverse health and behavioural outcomes such as body dysmorphia, eating disorders, and self-esteem problems [7]. Furthermore, exposure to harmful online content, particularly violent or sexually explicit material, can add stress to adolescents with genetic vulnerabilities, potentially triggering or worsening mental health problems [8].

## THE PROBLEM: PROBLEMATIC INTERNET USE

Problematic internet use (PIU), which is broadly referred to as preoccupation, loss of self-control over internet usage, withdrawal issues, negative impact on daily functioning, deception [9], and sexual-related online content addiction in particular, are areas of increasing scientific, health, and well-being concern [10]. Emerging evidence indicates that young people and individuals in LMICs are at the greatest risk of PIU [11]. Worldwide, one in three internet users are under the age of 18 [12], while 85% of adolescents owned a mobile phone by the age of 14 [13] and spent approximately 6.6 hours per day for non-school purposes [12]. Compared to high-income countries, where internet access is already widespread, LMICs are witnessing a rapid and dynamic expansion of their internet user base [12,13]. This growth is often driven by young people, who constitute a larger proportion of the population in these countries. They are more likely to gain access to uncensored and unsupervised online content, quickly adopt new technologies [14]. For example, the proliferation of digital health promotion tools in LMICs enables adolescents to access health-related information discreetly, providing both potential benefits and risks [15]. Furthermore, various organisational reports highlight that the lack of internet regulation in many LMICs leads to greater exposure to uncensored content, which can impact young people's mental health and social behaviours [14]. The increased use of the internet during the COVID-19 pandemic is also more pronounced in the LMICs which majorly manifested as excessive, maladaptive, or PIU [15]. This viewpoint emphasises the evidence connecting PIU with adolescent health and well-being and the challenges in early identification and treatment, aiming to trigger discussion and advance research and policy initiatives.

## WHY DO WE CARE ABOUT ADOLESCENT PIU

Adolescents' PIU, particularly in relation to sexual-related online content addiction (with reported pornography use ranging from 23–63% of adolescents aged between seven and 17 years old) has been increasing [16–18]. Exposure to sexual-related content addiction is linked to family and interpersonal relationship issues including relationship dysfunction, social isolation, low self-esteem, poor sleep quality, anxiety, depression, and reduced productivity [19,20]. Consequently, the deception, secrecy, and violations of trust associated with such addiction may shatter intimacy and personal connections [21,22]. This would result in a warped intimacy that often leads to separation and divorce and hampers any future healthy relationship [23,24].

## CHALLENGES OF IDENTIFYING ADOLESCENTS WITH PIU

Identifying adolescents with sexual-related online content addiction is challenging due to its sensitive nature. People are often reluctant to talk about it, and the physical and psychological signs are often subtle or hidden. Cultural, religious, and social stigmatisation often prevent adolescents in LMICs from seeking help for sexual-related PIU due to fear of judgment, moral condemnation, and lack of awareness about the issue [25]. This stigma can often lead adolescents to fear embarrassment or shame, leading them to avoid discouraging them from discussing their issues or seeking assistance. For instance, studies have highlighted that in various ethnic and cultural groups, there is a preference for dealing with problems within the family or through spiritual means rather than seeking professional mental health services. Adolescents may internalise their struggles or turn to family members and community support, which can be insufficient for addressing serious mental health concerns associated with PIU specific to sexual-related online content addiction [26]. Adolescents might face ridicule or dismissal from peers and adults who view their struggles as merely a lack of discipline. In countries with tight religious practices, the combination of strict religious norms and limited mental health services creates a challenging environment for adolescents to seek help [27]. Moreover, the cultural, religious, and social stigmatisation of the problem creates an atmosphere that does not promote treatment and prevention [28]. Studies have shown that public stigma towards addiction, particularly internet sex addiction, can result in internalised shame and guilt, further discouraging individuals from seeking the necessary treatment [29]. The perception of addiction as a moral failing rather than a medical condition perpetuates this stigma, making it harder for affected individuals to come forward and access support services [30]. As a result, seeking care and access to care, even when one recognises that own sexual behaviours are out of control, is a decision faced with further layers of barriers and limitations [25–27].

## THE ELEPHANT IN THE ROOM: GAPS IN ADDRESSING PIU

Adolescent sexual-related online content addiction has received insufficient attention from researchers and clinicians, particularly in resource-limited settings. To effectively address this issue, it is essential to implement community-based education programs, engage cultural and religious leaders to reduce stigma, expand mental health services, and develop culturally sensitive interventions tailored to adolescents' needs. While the recent introduction of compulsive sexual behaviour disorder diagnosis to the new ICD-11 could be a major milestone, fostering adolescents’ health-seeking behaviour requires a transformative approach. **Table 1** outlines the current resources available and identifies the gaps in addressing adolescent sexual-related online content addiction.

**Table 1:** Current resources available and the gaps in addressing adolescent sexual-related online content addiction

Aspect	What is available	Areas of improvement
Screening	No such practice, but screening tools are in the process of development and validation [31].	Development and implementation of routine screening programs in schools and youth service centres.
		Use multi-method approaches, including behavioural assessments.
		Developing and advertising an online platform can help develop adolescents' self-awareness in seeking health care services.
Diagnosis	Limited standardised diagnostic criteria.	Develop specific diagnostic criteria for problematic sexual content use.
	Existing diagnostic tools are often not specific to sexual content addiction [21]	Create validated screening tools for health service use.
Treatment	No or limited practices	Cognitive-behavioural therapy (CBT) tailored to sexual-related online content addiction.
	Lack of specialised treatment programs for adolescents [32].	Development and implementation of adolescent-specific treatment protocols.
Prevention approach	Basic parental controls and internet filters.	Enhance digital literacy programs for parents and adolescents.
	Awareness campaigns with limited reach.	Broaden the scope of awareness campaigns to reach more diverse populations.
		Expanding a community and school-based welcoming peer-to-pear talk adolescent and youth facilities.
		Continuous monitoring and evaluation mechanisms on the progress of in-placed interventions should be implemented.

## INTERVENTIONS IN REAL-LIFE SCENARIOS

Interventions to address adolescent internet addiction may require culturally and religiously adaptable versions for countries and regions with more conservative communities. Examples of such adaptable interventions include: 1) Tuko Pamoja Program in Kenya. This initiative integrates traditional storytelling and community gatherings to educate adolescents about safe internet use and the risks of digital media. It involves local elders and uses culturally relevant narratives to convey its message [33,34]. 2) Digital Shakti in India. An initiative aimed at empowering young women to use the internet safely. It collaborates with local NGOs and uses workshops conducted in local languages to educate participants about online safety and digital literacy [35]. 3) Internet Segura in Brazil. A government-led programme that works with schools and community organisations to promote safe internet practices. It incorporates local cultural elements and includes training sessions led by community members [32]. Effective interventions to address adolescent internet addiction, especially related to sexually explicit content, require a collaborative approach involving various stakeholders, including families, schools, health care providers, industry, and governments with clear roles and responsibilities. Each stakeholder plays a crucial role in implementing and sustaining the recommended strategies. Forming coalitions or working groups can further enhance coordination and resource sharing.

## RECOMMENDATIONS IN CONTEXT

Limiting PIU in general and sexual-related online content in particular needs appropriate measures.

1. Providing families with helpful guidance about their children’s internet and social media use and for families to monitor the contents that their children are accessing and how they are interacting with it. Studies highlighted the importance of parental mediation and monitoring in managing children's internet use, emphasising that guidance and active monitoring can reduce exposure to inappropriate content [36,37]. In regions where culture and religion play a significant role, involving religious teachings and leaders in the intervention can be beneficial. This might involve religious leaders and socially accepted people speaking about the importance of responsible internet use during sermons or community gatherings.

2. Empowering adolescents to be responsible online participants through active discussion and mutual understanding. Various sources have discussed strategies for empowering young people in this aspect, including the role of active discussion and education in fostering safe online behaviours, integrating digital citizenship lessons into school curricula, where educators can use real-life scenarios to teach students about the rights and responsibilities associated with digital interactions [38–41].

3. Encourage industries working on online sexual-related content to develop a tight protective mechanism for online access for inappropriate age [42]. A study highlighted the role of internet service providers in ensuring digital safety through protective mechanisms and corporate social responsibility initiatives [43].

4. Initiate countries to implement stringent legal framework is crucial for restricting access to online sites for adolescents [44]. Measures such as the Juvenile Protection Act, Internet Safety Zones, and the Real-Name System can effectively limit exposure to harmful online content and promote safer internet use among these age groups. For example, South Korea’s implementation of legal and restrictive frameworks serves as a real-world example of how countries can regulate access to inappropriate internet content to protect minors [45,46]. South Korea employs Server Name Indication (SNI) filtering, allowing internet service providers (ISPs) to block specific websites by monitoring the server names requested by users, thus preventing access to blacklisted sites without over-blocking other legal content. This approach aims to be more precise compared to traditional methods like Domain Name System (DNS) filtering, which can inadvertently block multiple sites sharing the same IP address. The Real Name Verification system also requires users to verify their identity before contributing content, which helps to trace posts back to specific individuals, encouraging self-censorship and compliance with regulations designed to prevent the spread of harmful content [45,46]. Furthermore, the ‘Safer Internet program’ is an initiative that aims to promote safer internet usage among children and adolescents across the European Union member states. It includes funding for hotlines to report illegal content, educational programmes, and the development of tools to filter and block inappropriate content [47,48].

5. Establishing community centres that can offer educational workshops, support groups, and safe internet access points can provide a supportive environment for adolescents. These centres can also serve as hubs for disseminating culturally relevant and adaptable information [49].

6. Promoting further research to explore the extent of the problem, diagnosis, treatment, and protection approaches.

7. Developing screening tools, books, and guidelines that help families and health professionals with early detection and treatment [42,50].

8. Finally, mechanisms for monitoring and evaluating the effectiveness of the recommended interventions such as changes in internet usage by adolescents and establishing feedback loops where stakeholders can report on the effectiveness of interventions and suggest improvement, should be in place.

## CONCLUSIONS

Addressing the multifactorial causes of mental health problems among adolescents requires a comprehensive approach. This approach should include monitoring and evaluating the effectiveness of interventions, fostering collaboration between stakeholders, and implementing culturally and religiously sensitive strategies. Families, schools, health care providers, industry, and governments must work together to develop and promote digital literacy, provide support and guidance, and establish protective mechanisms against inappropriate content. By integrating community-based education, empowering young people, enhancing digital safety measures, and promoting further research, we can mitigate the negative impacts of PIU and support the mental well-being of adolescents globally. These efforts are especially critical in LMICs where the challenges are most pronounced. Lastly, emphasising collaboration and clearly defining the roles of relevant stakeholders, we can also create a robust support system to address adolescent sexual-related online content addiction and promote healthier internet usage habits.

**Correspondence to:** Dr Abel Fekadu Dadi Menzies School of Health Research, Charles Darwin University Northern Territory Australia abel.dadi@menzies.edu.au.
